# A Case of Recurrent Dermatofibrosarcoma Protuberans With Fibrosarcomatous Transformation

**DOI:** 10.7759/cureus.89284

**Published:** 2025-08-03

**Authors:** Nadiim C Doyle, Lord Mvoula, Ali Raza, Raji Mohammed

**Affiliations:** 1 Surgery, St. George's University School of Medicine, True Blue, GRD; 2 Surgery, Lincoln Medical and Mental Health Center, Bronx, USA; 3 Surgical Oncology, New York City Health and Hospitals Corporation (NYC HHC) Lincoln, Bronx, USA; 4 Pathology and Laboratory Medicine, New York City Health and Hospitals Corporation (NYC HHC) Lincoln, Bronx, USA

**Keywords:** col1a1-pdgfrβ fusion protein, dermatofibrosarcoma protuberans, dfsp, dfsp-fs, fibrosarcomatous transformation, high mitotic count, oncogenic chromosomal translocation, soft tissue sarcoma

## Abstract

Dermatofibrosarcoma protuberans (DFSP) is a rare soft tissue sarcoma with a generally low risk for metastasis. Most individuals are diagnosed with this neoplasm in the third or fourth decade of life, with the growth usually being no larger than 5 cm in maximum diameter at the time of diagnosis. The majority of these patients are treated with wide local excision, with most experiencing no recurrence or metastasis. There are, however, cases of DFSP that are characterized by the presence of a high-grade fibrosarcomatous component, which represents a more ominous finding and exhibits a far more troubling course. To underscore the difference in the two presentations, we report a case of DFSP with fibrosarcomatous transformation in a 45-year-old man with two previous excisions. The size of the lesion, along with other characteristic findings, has been shown to worsen prognosis and increase risk of metastasis. Therefore, further research into the condition and therapeutic modalities to help patients is warranted.

## Introduction

Dermatofibrosarcoma protuberans (DFSP) is a type of soft tissue sarcoma with a high propensity for local relapse and a low risk for metastasis [1]. Age at diagnosis over 60, male sex, incidence of recurrence, tumor size > 3 cm, histologic grade III characterized by increased cellularity, cytologic atypia, and a high mitotic count (denoted as mitotic figures (MF) per high-power field (HPF)) are poor prognostic indicators for DFSP [2-4].

About 10%-20% of DFSP patients present with fibrosarcomatous transformation (DFSP-FS) [1,5]. DFSP-FS constituting more than 5% of the tumor volume carries a significantly high risk of recurrence (relative risk: 2.2, 95% confidence interval (CI): 1.7-2.9), metastasis (relative risk: 5.5, 95% CI: 4.3-7.0), and death (relative risk: 6.2, 95% CI: 5.0-7.8) [6]. Thus, DFSP is an uncommon soft tissue neoplasm with a good prognosis when seen in isolation, but when seen with fibrosarcomatous transformation, it is more worrisome [2].

Pathological differences between DFSP and DFSP-FS include atypical spindle cells, usually CD34 negative, high mitotic activity (median: 7 MF/10 HPF) arranged in a herringbone pattern, a higher proportion of cells containing t(17;22)(q22;q13), and increased incidence of microsatellite instability associated with p53 overexpression [1,5,7,8]. However, it is unclear whether necrosis should be considered a distinct feature of DFSP-FS [5].

In addition, DFSP-related tumors are generally small to moderate in size, while DFSP-FS tumors are larger at diagnosis. For example, Choong et al. reported significantly larger tumor size at diagnosis in DFSP-FS than in classical DFSP (6.8 versus 3.2 cm) [9]. Similarly, Li et al. reported mean tumor sizes of 10.6 ± 19.2 cm^2^ among patients with DFSP and 12.7 ± 17.7 cm^2^ in patients with DFSP-FS [10]. Based on the most extensive reported case series of 41 patients, the mean size of DFSP-FS is 4.8 cm, with 65% of the tumors measuring less than 5 cm [11]. By comparison, 84% of cases of DFSP were less than 5 cm in size, 13% were between 5 and 10 cm, and only 3% were over 10 cm [12]. Furthermore, ~77% of tumors remained superficial, with the remainder having some degree of invasion of deeper structures [12]. Fiore et al. reported median tumor sizes of 2 cm in patients with primary (interquartile range (IQR): 2-3) or recurrent (IQR: 1.5-4) DFSP [13].

Although DFSP-FS is most commonly seen in the trunk (51%-62%), followed by the limbs (18%-32%) and head and neck region (10%-15%) [11,14,15], rare cases have also been reported in the breast [16] and the site of breast implants [17]. DFSP-FS is typically managed with surgery with wide excision (margins ≥ 2 cm) associated with significantly lower rates of recurrence [8].

This case report features a patient with DFSP-FS in whom high-grade cancer was of impressive size (18 × 25 cm), nearly four times the average size. This report highlights the patient's course to determine risk factors leading to accelerated growth, compared to similar cases, and to assess prognostic factors, specifically, as they relate to recurrence in previously treated individuals. The patient was asked for permission to include clinical photographs of the mass in this report and kindly agreed.

## Case presentation

Initial presentation

**Figure 1:**
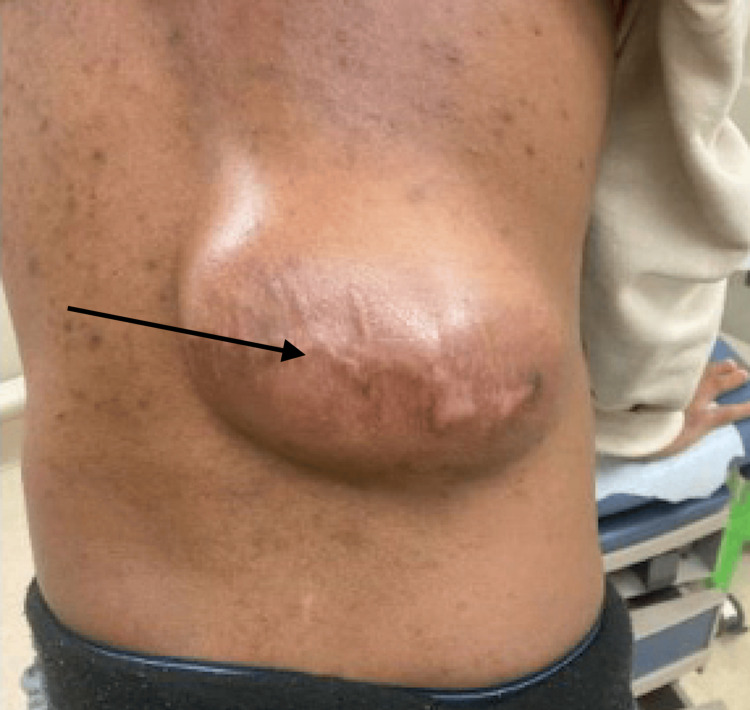
Photographs of the Mass on the Patient's Back Photographs of the mass (black arrow) on the right lumbar portion of the patient's back as they appeared on the day of his presentation to the emergency department. Scarring from previous excisions can be appreciated overlying the mass.

A 45-year-old man from Ghana presented to the emergency department after falling on his back, causing pain from an existing mass on his back. His past surgical history was significant for resections of a similar back mass in the exact location twice, once eight years ago and again two years ago, for which the pathology results were not available. The patient reported that the back mass had grown slowly over the past 10 years, but considerable growth had occurred over the past year. He had no recent trauma to the site and did not report other malignancies or radiation exposure. The appearance of the mass on presentation to the emergency department is shown in Figure 1.

On physical examination, an 18 × 12 cm mass was noted in the right mid-back to lumbar region, extending laterally, with overlying scarring from previous excisions that appeared mildly erythematous, indicating possible inflammation secondary to recent trauma. On palpation, the mass was fixed to the underlying structures. The medial aspect of the mass was firm and non-tender, while the lateral aspect was moderately tender and soft. The patient reported mild pain given his recent fall, but he denied any weight loss or local mass-related symptoms. Range of motion was normal, gait was strong and steady, and lower extremity strength was normal bilaterally.

Imaging findings

**Figure 2:**
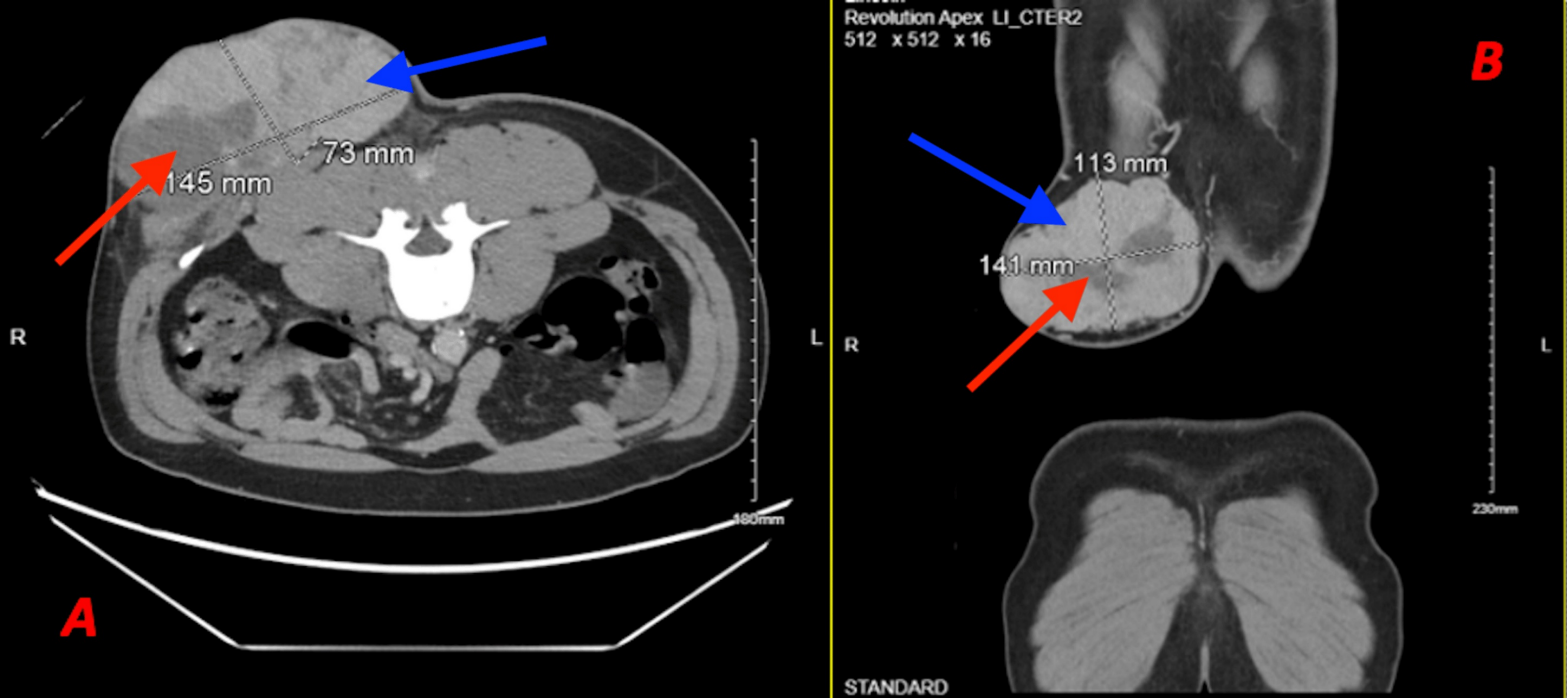
CT Scan Taken on the Day of Presentation Axial (A) and coronal (B) CT scan sections, which showed the size of the mass on the patient's back on the day of his presentation to the emergency department. Two distinct densities are appreciable within the tumor, denoted by blue (less dense) and red (more dense) arrows. CT: computed tomography

The patient underwent a chest/abdomen computed tomography (CT) scan, which showed a large, hypervascular soft tissue mass within the right back and flank, with further review of imaging reporting abutment and superficial invasion of the erector muscles and possibly the external oblique. On imaging, the mass featured two characteristically distinct areas, a denser area that seemed to predominate laterally and a less dense area medially, identifiable in Figure 2. The review did not show any evidence of lymphadenopathy or distant disease.

Intraoperative ultrasound was used at the time of biopsy, which showed two different densities at the lateral aspect of the lesion, consistent with what had previously been seen on CT imaging. A 4 cm long × 1 cm wide ellipse was drawn over the tumor, and a 4 cm long × 1 cm wide × 2 cm deep sample was excised with two distinct gross appearances. The tumor's medial portion appeared yellow and tan, while the lateral portion appeared hypervascular, hemorrhagic, and dark burgundy, suggesting an area of fibrosarcomatous transformation.

Histopathologic findings

**Figure 3:**
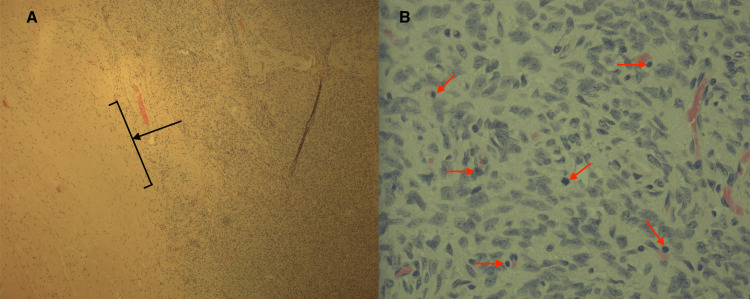
Histologic Sections From the Mass of the Patient's Back A: H&E-stained slide showing a portion of DFSP taken from the excised mass on the patient's right lumbar back. Necrosis can be appreciated at the lateral portion of the sample (black arrow with bracket). B: H&E-stained DFSP at 400×. At this magnification, one can identify spindle-shaped cells arranged in a storiform and fascicular pattern, as well as several mitotic bodies (red arrows). H&E: hematoxylin and eosin, DFSP: dermatofibrosarcoma protuberans

Histopathologic review of the biopsy specimens showed a predominating fibrosarcomatous component, largely in the lateral aspect of the tumor, composed of monomorphic spindle cell proliferation arranged in a storiform and vesicular pattern involving the dermis and subcutis with increased cellularity and a high mitotic count (>10 MF/HPF), as seen in Figure 3. The tumor was, thus, staged clinically as T4NxMx.

Treatment

**Figure 4:**
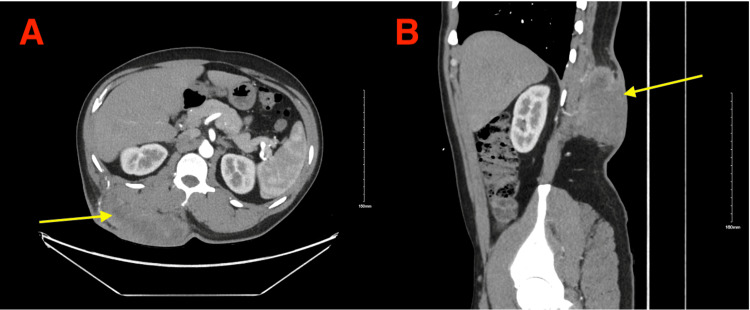
CT Scans Taken Following Radiation Therapy Post-radiation therapy axial (A) and sagittal (B) CT sections showing the extent of the mass (yellow arrows) in the right lumbar region of the back. CT: computed tomography

It was recommended to the patient that he undergo preoperative external beam radiation therapy before surgical excision. Subsequently, the patient received radiation therapy of 5,000 cGy to the mass and margins at 200 cGy per fraction per day. Radiation therapy was completed in 25 treatment sessions over five weeks. CT imaging taken after completion of radiation therapy, as seen in Figure 4, showed a slight decrease in the size of the tumor.

**Figure 5:**
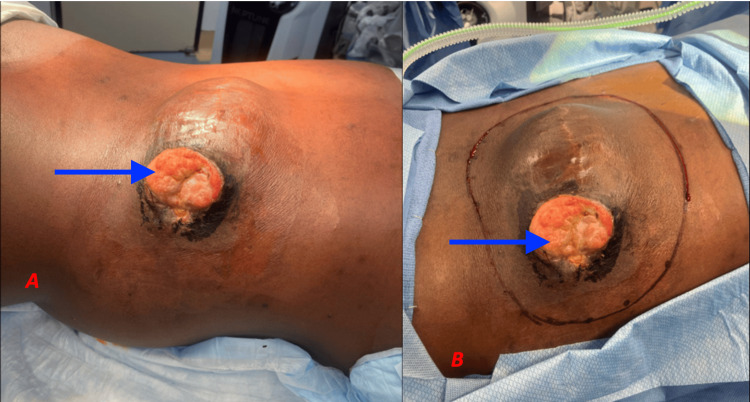
Photographs of the Lesion Prior to Excision Photographs of the mass on the patient's back immediately prior to wide radical excision. One can appreciate some necrosis and skin changes on the surface of the mass, associated with radiation therapy, as well as the ulcer (blue arrow), which formed at the biopsy site.

After post-radiation therapy recovery, the patient was reassessed, and the tumor appeared slightly smaller and contracted; an ulcer had formed at the incisional biopsy site but remained uninfected (Figure 5). The patient was counseled and underwent wide radical resection.

**Figure 6:**
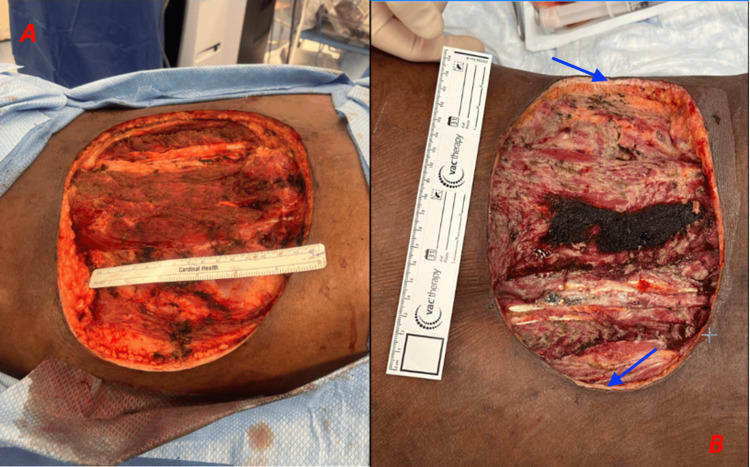
Postoperative Photographs of the Patient's Back Wound Photographs of the right lumbar portion of the patient's back taken postoperatively. Photograph A was taken immediately postoperatively, highlighting the necessity to remove the mass and portions of the overlying latissimus muscle, erector spinae, and external oblique to attain cancer-free margins. Photograph B was taken three days postoperatively, showing that the wound appeared healthy, with some granulation tissue around the edges (blue arrows).

The surgical procedure involved resection of the mass along with portions of the overlying latissimus muscle, erector spinae, and external oblique (Figure 6). The dimensions of the resected sarcoma measured 18 × 12 cm in the craniocaudal plane and 2 cm circumferential margin, while the primary resection measured 18 cm in the craniocaudal plane and 25 cm in the diagonal plane. Despite a grossly negative initial deep margin, a second deep-margin specimen was obtained and measured 5 × 3 cm. The pathology results reported DFSP with a high mitotic rate (~5 MF/10 HPF) and negative margins, the closest being the deep margin at 4-5 mm. Wound care was performed with serial wound vacuum-assisted closure (VAC) dressing changes.

**Figure 7:**
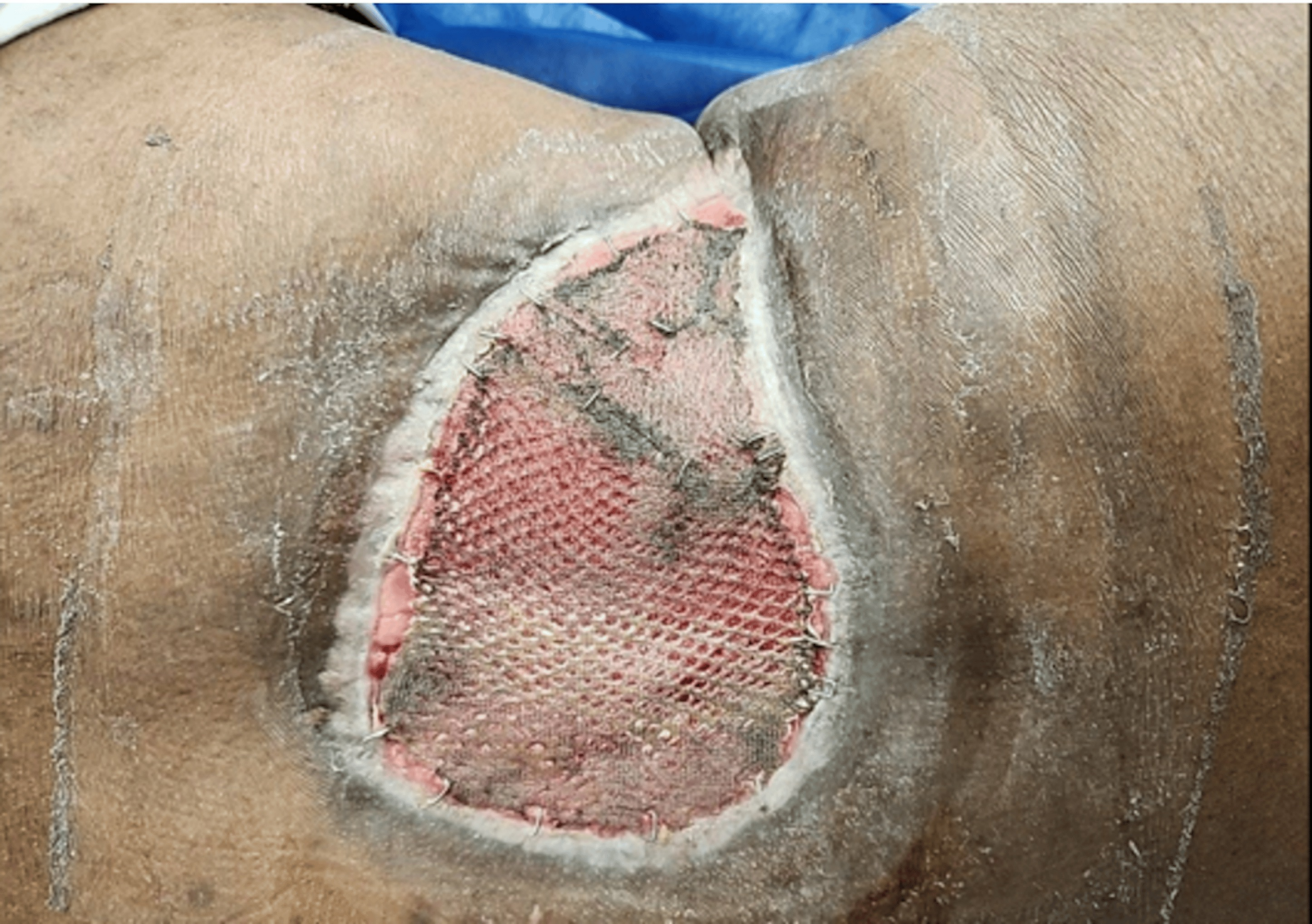
Photograph of the Patient's Wound Following Skin Grafting Photograph of the wound on the right lumbar portion of the patient's back taken after successful skin grafting carried out by the plastic surgery service.

The patient was referred to the plastic surgery service for reconstruction. He underwent split-thickness skin grafting without complications (Figure 7). His three-month follow-up CT imaging revealed no evidence of distant disease.

## Discussion

Comparison with other cases

While several case reports on DFSP-FS with unique features and sites have been reported, we could only identify two reported cases of extremely large DFSP-FS [18,19]. Lee et al. reported a case of extremely large DFSP-FS (the largest nodule measured 16 × 15 × 7 cm) on the left shoulder, which started following a minor work-related injury 12 years before presentation [18]. While traumatic injuries have been hypothesized as a risk factor for DFSP and DFSP-FS [18], as is suspected in our patient, a case of extremely large DFSP-FS measuring 30 cm in the occipital region of the head has been recently reported in a patient without any history of traumatic injury [19]. Nonetheless, several overlaps stand out in these cases and ours: the tumor size showed massive growth within a couple of months after a prolonged period of slow growth, the tumor was ulcerated and necrotic, and all three cases required a wide excision to achieve DFSP-free margins, requiring free flap and/or split-thickness skin graft for reconstruction. These symptoms and treatment courses are consistent with average-sized DFSP-FS [20,21]. The treatment strategy represents the current standard of care in managing DFSP, which is generally wide local excision with a 2-3 cm margin, with adjuvant radiation therapy as an option either before or after resection [22].

Prognosis and recurrence risk

Previous studies indicate that patients with DFSP-FS will require long-term monitoring for disease recurrence. The most extensive single-institutional study on DFSP by Bowne et al. reported poorer five-year recurrence-free survival rates for DFSP-FS (28% versus 81% in DFSP), and overall relapse was 16% in DFSP and 52% in DFSP-FS [12]. Analysis of these cases found that two reliable indicators of future recurrence were intermediate histologic grade and close or positive margins [12]. Another study by Lyu and Wang assessed post-resection recurrence rates in patients diagnosed with both primary and recurrent DFSP and DFSP-FS between three and 36 months and reported DFSP recurrence in 17.1% of primary cases, whereas in recurrent cases, further recurrence developed in 37.9% of patients [23]. Differences in recurrence rate between cases of DFSP and DFSP-FS were even more stark. Patients with DFSP experienced an overall recurrence rate of 20.3%, while DFSP-FS recurred in 54.6% of patients [23].

Further, about 2% of overall patients in the study by Lyu and Wang developed metastatic disease, with two-thirds diagnosed with DFSP-FS [23]. In an earlier study, Fiore et al. showed that patients with DFSP-FS accounted for 20% of cases that developed metastasis within 10 years (2/5), although they only represented about 3% of their sample size [13]. Furthermore, of the five patients who eventually developed metastasis, four had presented with recurrent cases of DFSP following excision more than 10 years prior [13].

Follow-up strategies

Although our patient had no signs of metastatic disease or recurrence at three-month follow-up, a prolonged monitoring strategy is necessary. Given the lack of standardized follow-up protocol for DFSP, some clinicians recommend ultrasound monitoring every six months for local recurrences and chest CT scan every 12 months for the first five years due to the high risk of lung metastasis [24]. Other experts have recommended even closer follow-up, such as Li et al., who suggest examination every three months for the first postoperative year and every six months thereafter, with ultrasound or MRI used to assess for soft tissue tumor recurrence and chest X-ray or CT to assess for pulmonary metastasis [10]. While their study was only able to report on follow-up of six years at the time of publication, they note that systematic reviews have shown a mean recurrence time of 5.7 years, indicating a need for longer monitoring.

Emerging therapies and molecular insights

However, recent breakthroughs in cancer therapeutics, particularly for irresectable and aggressive cases, may be effective in maintaining long-term remission. For instance, Rutkowski et al. [25] assessed clinical response to imatinib mesylate among 31 patients with locally advanced, inoperable, or metastatic DFSP with cytogenetically proven presence of the COL1a1-PDGFRβ fusion protein, as its mechanism of action involves the inhibition of autophosphorylation and activation of multiple proteins by tyrosine kinases [26]. In this cohort, 16 patients were diagnosed with DFSP-FS, and 15 presented with metastasis, all of whom had lesions with areas of fibrosarcomatous transformation. Imatinib elicited a positive response in tumor burden, with the study producing a five-year progression-free survival (PFS) of 58% and a five-year overall survival (OS) of 64% [25]. Further analysis of these figures underscored the tragic consequence of fibrosarcomatous transformation, as shorter PFS and OS were strongly correlated with DFSP-FS and metastatic disease. Ultimately, the five-year PFS for classic DFSP was 93% and 33% for DFSP-FS [25].

## Conclusions

This patient's presentation aligns with observations noted in previous literature, although several unique factors are present. Firstly, the lesion excised from the patient measured 18 × 25 cm, significantly larger than the average size of dermatofibrosarcoma, which is typically under 5 cm. Histopathological analysis of a biopsy from this mass revealed necrosis, an exceedingly rare finding in dermatofibrosarcoma. Furthermore, three indicators of poor prognosis, namely, fibrosarcomatous transformation, recurrence, and the initial size of the lesion, were noted.

This report highlights the crucial importance of early and accurate diagnosis, as well as complete and meticulous resection of dermatofibrosarcoma, to prevent recurrence and the potential for aggressive transformation into DFSP-FS. It also highlights the necessity for closer and more prolonged follow-up for patients after resection, given that recurrence rates are relatively high and are often linked to a more rapid progression and increased malignant potential.
